# Inductive Heating of Ceramic Matrix Composites (CMC) for High-Temperature Applications

**DOI:** 10.3390/ma17102175

**Published:** 2024-05-07

**Authors:** Alexander Hackert, Jonas H. M. Stiller, Johannes Winhard, Václav Kotlan, Daisy Nestler

**Affiliations:** 1 Professorship Forming Technology, Department of Mechanical Engineering, Chemnitz University of Technology, 09107 Chemnitz, Germany; 2 Funded Research Group Textile Plastic Composites and Hybrid Compounds, Department of Mechanical Engineering, Chemnitz University of Technology, 09107 Chemnitz, Germany; jonas.stiller@mb.tu-chemnitz.de (J.H.M.S.); johannes.winhard@mb.tu-chemnitz.de (J.W.); daisy.nestler@mb.tu-chemnitz.de (D.N.); 3 Regional Innovation Centre for Electrical Engineering, University of West Bohemia, 306 14 Pilsen, Czech Republic; vkotlan@fel.zcu.cz

**Keywords:** ceramic matrix composite, induction heating, carbon fiber, silicon carbide, composite, high temperature application

## Abstract

The inductive heating of a CMC susceptor for industrial applications can generate very high process temperatures. Thus, the behavior of a silicon carbide-based matrix with carbon-fiber-reinforced carbon (C/C-SiC) as a susceptor is investigated. Specifically, the influence of fiber length and the distribution of carbon fibers in the composite were investigated to find out the best parameters for the most efficient heating. For a multi-factorial set of requirements with a combination of filling levels and fiber lengths, a theoretical correlation of the material structure can be used as part of a digital model. Multi-physical simulation was performed to study the behavior of an alternating magnetic field generated by an inducing coil. The simulation results were verified by practical tests. It is shown that the inductive heating of a C/C-SiC susceptor can reach very high temperatures in a particularly fast and efficient way without oxidizing if it is ensured that a silicon carbide-based matrix completely encloses the fibers.

## 1. Introduction

Industrial high-temperature applications have relied on fossil energy sources with a low level of efficiency. As a result, there is great interest in researching efficient and sustainable alternatives. Induction heating technologies are energy efficient and can be utilized from renewable energy sources [1,2,3]. In the last twenty years, electrically and thermally conductive susceptors have become essential for heating a broad spectrum of non-conductive materials, primarily ceramics and polymers. They were initially made of metals such as tungsten, molybdenum, stainless steel, and graphite, mainly in a tubular arrangement. Unfortunately, some of these materials exhibited certain disadvantages, the principal of which was their oxidation in air at higher temperatures. Their utilization was, therefore, restricted to inert environments. However, in some applications, even graphite may prove unsuitable due to the danger of contaminating the heated material. In order to generate high process temperatures by induction, carbon can be inductively heated to very high temperatures. To directly exploit the efficiency advantages of induction, a special feature is used. Carbon has very large penetration depths of 2 to 10 mm at frequencies between 100 kHz and 2 MHz [4]. Therefore, an alternating magnetic field can only couple to carbon fibers (CF) with a maximum diameter of 5 to 9 µm if they allow the current to flow or form a network [5]. For this condition to occur, there must be a sufficient quantity of fibers present, with a minimum fiber length, and they must form agglomerates that allow current flow [6]. In addition, the very small volume of the CF means that there are virtually no thermal conduction losses, resulting in particularly rapid heating. The SiC-coating protects the carbon fibers from oxidation and damage. However, due to the rapid oxidation of the fibers in ambient air, a high temperature resistant matrix is required to protect the fibers from contact with oxygen [7]. One such ceramic is silicon carbide (SiC), which is particularly suitable as a susceptor material due to its application temperature of over 1800 °C [8,9]. Park et al. even demonstrated the thermomechanical stability of a Cf/SiC that was produced with a precursor infiltration and pyrolysis for up to 2000 °C [10].

The so-called ceramic matrix composites (CMC), commonly known as fiber-reinforced ceramics or fiber ceramics, have their origin in a CF-reinforced carbon matrix (C/C) in the aerospace sector and were transferred to friction applications for, e.g., high-performance vehicles and elevators [11,12]. C/C parts are broadly used as aircraft brakes, but lack braking performance below 200 °C and in wet conditions [13,14]. Therefore, protection by silicon carbide was introduced to protect C/C from oxidation, which led to the new material consisting of CF and a dual-phase matrix of carbon and SiC [15] that also improved the coefficient of friction in tribological applications.

There are three established production processes to integrate CF into a SiC: chemical vapor infiltration (CVI), precursor infiltration and pyrolysis (PIP), which is also called liquid polymer infiltration (LPI), and liquid silicon infiltration (LSI), which is used for the production of automotive brake discs [16]. The CVI and PIP/LPI-processes are mainly used for the production of continuously reinforced parts, whereas the LSI process is also used for processing short fiber reinforced parts [17,18,19]. The LSI process has three main steps: (1) shaping of the CF reinforced plastic (CFRP) part, where multiple processes like pressing, RTM, or filament winding can be used; (2) pyrolysis to C/C to detach all non-carbons and form a crack network; and (3) liquid silicon infiltration into the cracks to form C/C-SiC. Typical matrix materials have a rather high char yield and are dimensionally stable during pyrolysis [20,21]. Therefore, phenolic resins like Novolak and resoles or thermoplastics like PEEK are used, as are matrix polymers [22,23]. The latest developments have shown that, in particular, the shaping process can be drastically improved in terms of productivity and reproducibility as well as in freedom of design. Those production processes include thermoset injection molding with Novolak for large-scale production that enables the integration of 6 mm CF short fiber bundles [24,25], additive manufacturing with PEEK [26,27], the pultrusion process of unidirectional reinforced profiles with a fiber volume content of up to 66% [28], and the use of automated fiber placement [29].

Therefore, carbon in a two-phase matrix of carbon and silicon carbide (C/C-SiC) is used to investigate heating behavior. The carbon and the carbon fibers should be completely enclosed by the silicon carbide so that no oxygen can penetrate and cause oxidation at high temperatures. The influence of the fiber length on the fiber distribution and the inductive heating behavior of the C/C-SiC composite is discussed. We investigated whether the fiber length leads to agglomeration and whether the resulting networks are conducive to the coupling of the alternating magnetic field. Light microscopy and SEM images are used to analyze the damage mechanisms. They will show how the embedded fibers behave due to the high temperatures. The damage should allow conclusions to be drawn about the processes during inductive heating. The evolution of the loss of structural integrity in the matrix and its propagation will be analyzed. Fiber length comparisons will be made for the 50% filled version. The objective of this paper is to evaluate the behavior of C/C-SiC during induction heating.

## 2. Materials and Experimental Methods

### 2.1. Materials and Manufacturing

**Figure 1:**
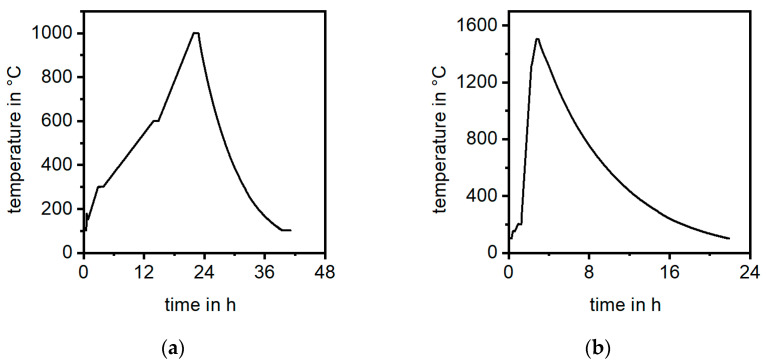
Temperature–time profiles of (**a**) the pyrolysis process; (**b**) the liquid silicon infiltration process.

An established manufacturing process was used to produce the sample material. A commercial phenolic resin based on a Novolak with dropping point of 121 °C with the hardener hexamethylenetetramine (Prefere Resins Germany GmbH, Erkner, Germany) was homogeneously mixed in the twin-screw extruder ERMAFA 2.30 (ERMAFA Maschinen- und Anlagenbau GmbH, Chemnitz, Germany), together with zinc stearate (Baerlocher GmbH, Duisburg, Germany) as the lubricant and 6 mm Sigrafil chopped carbon fibers that are based on a 50 k tow (SGL, Wiesbaden, Germany). In this way, 10 kg/h of injection molding pellets can be produced. The material was used in thermoset injection molding to manufacture dog-bone-shaped tensile samples using a KM-250-1400-C2 (KraussMaffei AG, Munich, Germany) injection machine equipped with a thermoset plasticization unit. Because only the middle part of the samples is used for flexural testing, both ends of the dog bone sample were cut and used in this work. Those parts were then pyrolyzed at 1000 °C in an Argon atmosphere (Figure 1a) and infiltrated with liquid silicon (Figure 1b) at up to 1600 °C in vacuum in two separate furnace processes, both using the FCT-FS W 315/800-2400-PS (FCT Anlagenbau GmbH, Sonneberg, Germany).

Two variants of samples were produced, the first with a mass share of 50% of CF, with an average length of approx. 250 µm [17], and the other with 40% of short CF with additional 10 weight-% chopped 6 mm CF bundles, also resulting in a total mass share of 50%. They were called CF40+10 and CF50, respectively.

In addition to its excellent mechanical properties, the material used was also well suited for inductive heating processes. The ceramic matrix is particularly temperature resistant, and the carbon fibers contained therein can be inductively heated particularly well if networks have been formed by agglomeration. This is especially the case if the fibers are long enough. In [24], fiber lengths of about 6 mm could be processed by injection molding. Previously, they had to be much shorter, because the system conditions, such as the screw conveyor or narrow hot runners, allowed only short fibers. This study will show that the difference is critical for inductive heating. Therefore, samples with short and long fibers will be compared.

### 2.2. Experimental Simulations

**Figure 2:**
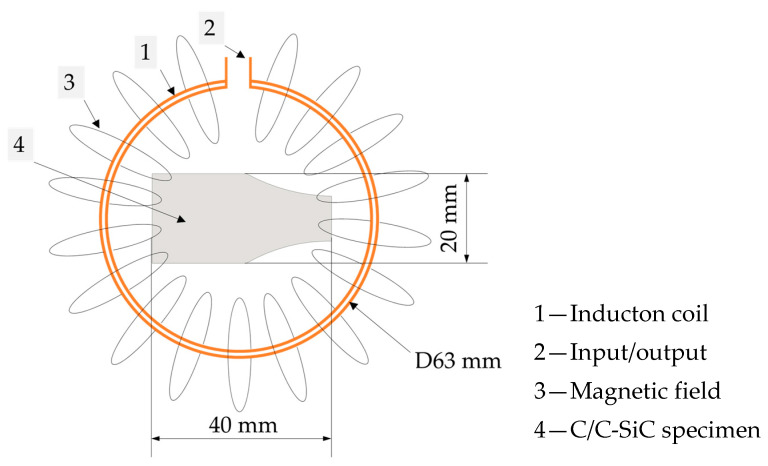
Induction setup with C/C-SiC specimen.

The material composition of the test series was created in the database using a simulation model. The test specimen was modeled for this purpose. It is a rectangular, flat body with the dimensions shown in Figure 2. In order to clearly assign the properties in the simulation, the areas of the carbon fibers, the porous carbon structure, and the silicon carbide were separated in the CAD model. The assignment was undertaken using the material library of the COMSOL Multiphysics simulation software. In addition, the specimen was placed in the center of a ring inductor, so that a quasi-2D state of the experimental situation could be mapped. For the simple ring inductor, the magnetic field propagation is known. It is centered in the interior and is strongest there.

The simulation was based on the same boundary conditions that would be used in the experiment. It was assumed that carbon has very good inductive heat ability in the mid-frequency range due to the particularly high penetration depth of the magnetic field. To comprehend the thermal effects induced by electromagnetic waves, this study integrated numerical simulations and experimental validations. Finite Element (FE) simulations, conducted using COMSOL Multiphysics software, offer detailed insights into the electromagnetic and thermal dynamics within the carbon fiber structures at the C/C SiC compound. These simulations enable the analysis of electromagnetic field distribution and resulting temperature profiles within the carbon fibers. Complementing these simulations, experimental investigations were carried out using a simple designed coil setup.

### 2.3. Experimental Setup

**Figure 3:**
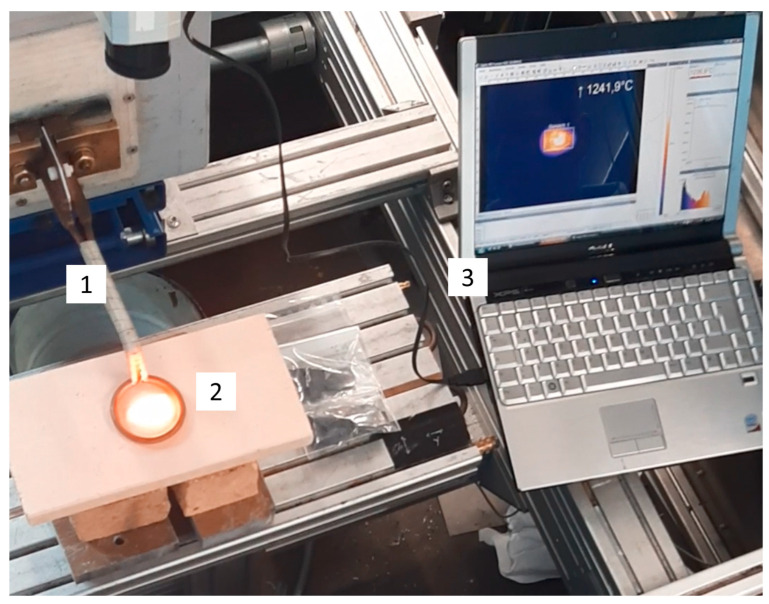
Experimental setup; 1—induction coil; 2—C/C SiC specimen; 3—temperature distribution.

As in the theoretical preliminary considerations, an experimental setup was chosen that allowed a simplification to two-dimensional results. For this purpose, a ring inductor consisting of a copper coil with a constant flow of cooling water was made. The ring inductor, with the dimensions proposed in the preliminary studies, was protected by a heat-resistant fabric tape in the feeding area. The experimental setup is shown in Figure 3.

The inductor installed in this way was driven by a medium-frequency generator (eldec HFG 50) with an external resonant circuit, which can deliver up to 50 kW of power for frequencies between 100 kHz and 1 MHz. The test setup also included a switch for pulsed operation during the run-in phase. During the actual tests, the generator was operated continuously. When the maximum temperature was reached, the system was able to switch off directly. A stepped output was used. The corresponding frequencies for use with the inductor coil used are assigned in Table 1.

In addition, the penetration depths of carbon were assigned to the different frequency and power ranges [2]. In general, the penetration depth of carbon is very high. It decreased as the frequency of the alternating magnetic field increased. For the dimensions of the samples to be examined, frequency ranges of 235 kHz and higher led to the full utilization of the penetration depth and, correspondingly, more effective results.

An optical thermal camera (Optis) was used for temperature monitoring. Its measuring ranges could be used variably. The temperature range between 450 °C and 1800 °C was used. During the experiments, certain areas of the experimental setup could be monitored and recorded using control software (Optris PIX Connect 3.12.3079.0). In this case, the two-dimensional sample geometry was defined as the monitoring pattern and the maximum temperature was recorded. In addition to the optical temperature distribution, the temperature curve over time was recorded. The maximum duration of a recording was set to <1 min.

### 2.4. Experimental Visual Investigations

The fiber orientation was visualized by longitudinal cuts through the samples using a digital light microscope, SmartZoom (Carl Zeiss AG, Oberkochen, Germany), with its stitching function to combine single images into one larger one. The fiber orientation was analyzed with imageJ [30] using the orientationJ plugin with its analysis and distribution functions. Finally, the cross-sections of the heated samples were investigated via scanning electron microscopy (SEM) using the ZEISS Evo 15 (Carl Zeiss AG, Oberkochen, Germany) with combined EDX-scan QUANTAX CrystAlign 200 (Bruker, Billerica, MA, USA).

## 3. Results and Discussion

This section analyzes the behavior of C/C-SiC composites when exposed to an alternating induction magnetic field. The relationships between the achievable temperature and the required generator power are presented. Two versions of the C/C-SiC samples are distinguished. The evolution of the temperature rise in the device is analyzed. The changes in the mechanical structure of the composite are compared using SEM images.

### 3.1. Experimental Results

**Figure 4:**
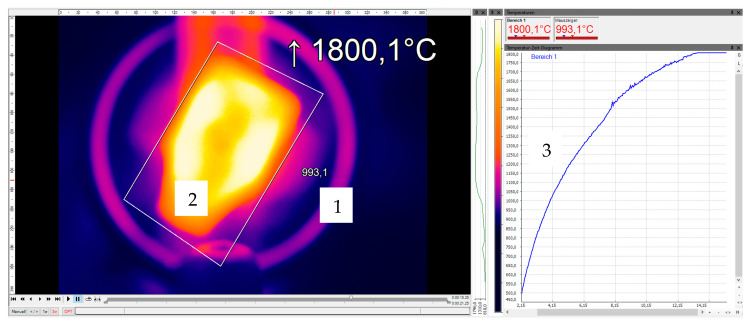
Measurement results: 1—induction coil, 2—C/C SiC specimen, 3—temperature distribution.

The results show that a C/C-SiC composite can be inductively heated. The process parameters for the test series were implemented. A very good heat ability could be established for all specimens. In all experiments, the frequency of the electromagnetic field, the coil current, and the maximum coil temperature were roughly the same. The measured maximum C/C-SiC temperatures were in a range of 930 ≤ T_max_ ≤ 1800 (°C). The heating rate was varied throughout the test series (Figure 4).

**Figure 5:**
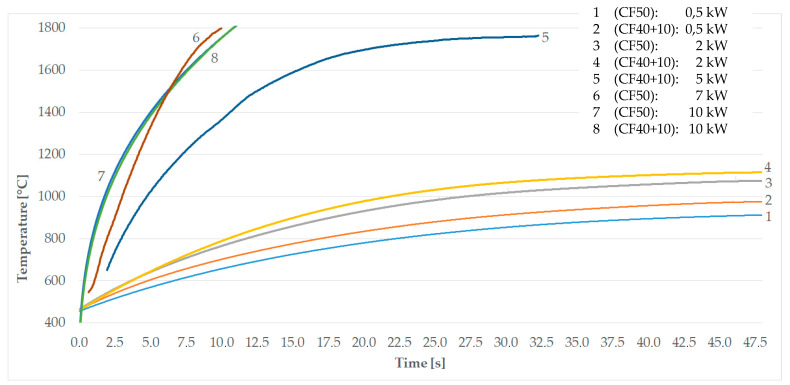
Behavior of samples at different generator outputs; comparison of different filling levels.

The length of the carbon fibers had an influence on the heat ability of each sample. The hypothesis that the SiC encapsulates the carbon fibers and protects them from oxygen ingress was also investigated. For this purpose, an experiment in which the samples were annealed and reached the highest temperature is examined in more detail. First, the results were evaluated using the temperature–time curve. Power ranges from 0.5 to 10 kW are shown. Figure 5 compares the different curves.

Samples with two different filling levels were used (CF50, CF40+10). In the first, the carbon content was 50%. The second variant contained 10% carbon fiber and 40% carbon. Performance levels 1 to 4 and 7 and 8 were tested with both variants. CF40+10 samples were used for performance level 5 and CF50 samples were used for performance level 6.

Reproducible heating rate behavior can be seen at the lower power levels. The higher power levels show significantly higher heating rates. The results confirm the assumption that the penetration depth is large enough at a certain frequency range and that more efficient results can be achieved. As suspected, the efficiency increases significantly from pass number six and the heating rates improve drastically. The influence of the long carbon fibers, which have formed networks through agglomeration, no longer comes into play. The alternating magnetic field acts completely on the carbon parts. The values obtained are summarized in Table 2.

### 3.2. Fiber Orientation

**Figure 6:**
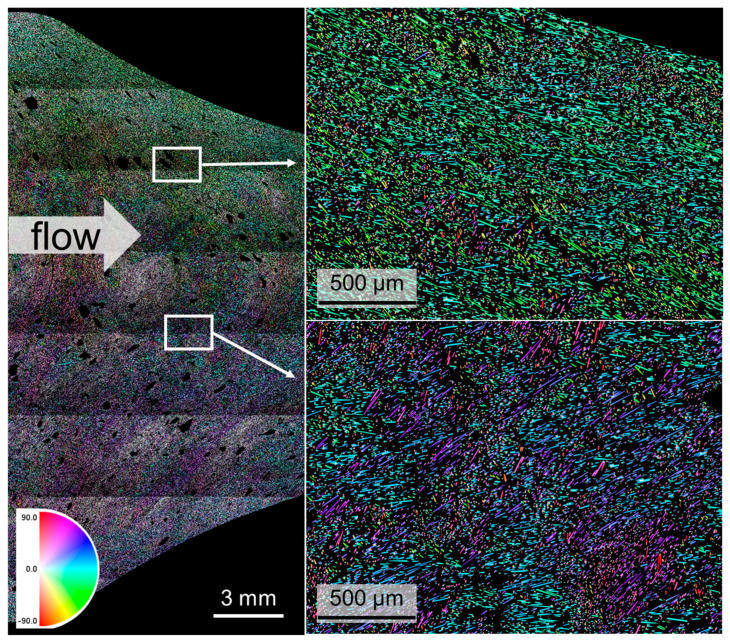
Stitched digital light microstructural image of a CF50 sample in CFRP state with magnification of two areas. The color code represents the fiber orientation.

The fiber orientation of both variants is totally different. The CF50 contains only very short CF, and the CF40+10 variant contains an additional 6 mm of chopped CF. In Figure 6, the CF50 sample is shown in the CFRP state with its fiber orientation and two local magnified areas in the center and at an edge of the sample. In the edge area, the fibers are mostly aligned parallel to the edge that equals the direction of flow, and in the interior the orientation is transverse to the flow. In general, the fibers are aligned tangentially on a half-circle that moves with the flow. Due to the high fiber share of 50% in mass, the CF are very close together.

**Figure 7:**
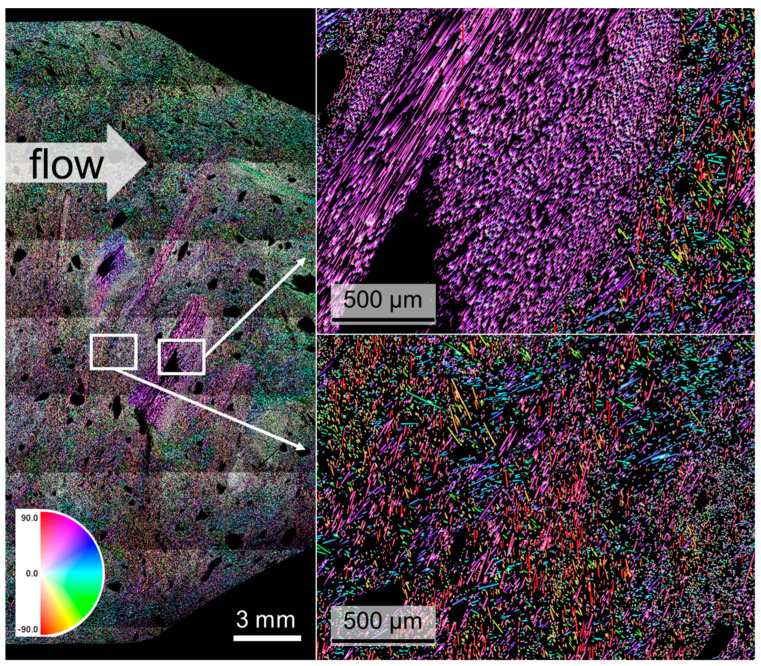
Stitched digital light microstructural image of a CF40+10 sample in CFRP state with magnification of two areas. The color code represents the fiber orientation.

The CF40+10 in CFRP state sample is displayed in Figure 7. The general alignment of the short fibers is the same compared to the CF50 sample, with the tangential fiber orientation in the core area and edge-parallel in the outer area. Conversely, the chopped fiber bundles do not correspond in their orientation to the alignment of the short fibers. Additionally, on the inside of those bundles, some dry sections that were not infiltrated by the resin in the injection molding process are visible. Also, the amount of porosity, resulting from the polycondensation crosslinking reaction of the Novolak with hexamethylenetetramine with the separation of ammonia, is higher than in CF50. There are also more and larger pores in the core than at the edges.

### 3.3. Post-Heating Analysis

**Figure 8:**
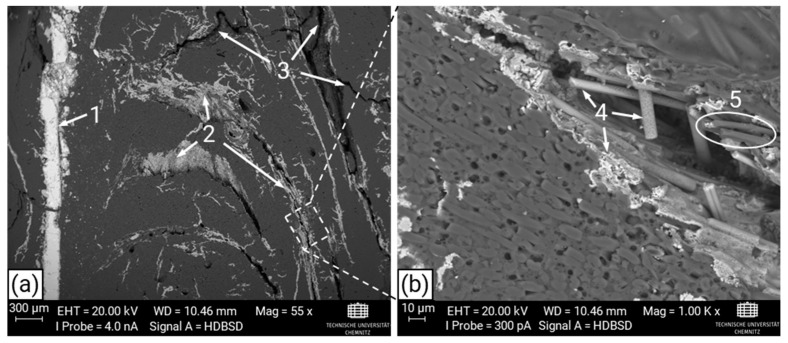
Cross-section of specimen CF50 imaged via back-scatter detector (BSD) with dark areas showing the carbon matrix and carbon fibers, as well as the silicon and silicon carbide appearing bright: (**a**) Overview of the specimen’s cross section with (1) pure silicon residues of the liquid silicon infiltration; (2) crack surfaces caused by pyrolysis and transformed carbon matrix and fibers with silicon to SiC; (3) cracks without infiltrated silicon. (**b**) Enlarged section of (**a**) with (4) SiC rich area and (5) C-fibers free of silicon.

Images of the cross-section of the CF50 specimen via SEM (Figure 8) show successful silicon infiltration. To distinguish between the carbon-based material (matrix and fibers appear dark) and silicon, as well as SiC, which appeared bright, the back-scatter detector was used for material contrast. Cracks exhibiting a wide spectrum of sizes are visible in Figure 8a. Although bright areas indicate silicon infiltration, cracks devoid of silicon traces are also present. These are attributed to internal cracks resulting from pyrolysis, inaccessible from the specimen’s surface, occurring during inductive heating or cross-sectional polishing. Consequently, silicon could not reach these exposed crack surfaces or the cracks formed post-infiltration. Additionally, fiber reactions with silicon (Figure 8b) are evident, with only a few fibers lacking silicon contact, likely due to their exposure during polishing and subsequent embedding within the matrix during silicon infiltration.

## 4. Discussion

Several studies have investigated the behavior of materials under the influence of electromagnetic fields [1,2,3,31]. The study results show that the induction heating of a C/C-SiC is excellent. Extremely high temperatures are achieved within very short cycle times. It can be seen that process temperatures in the range of about 1000 °C can be achieved even at low power levels. In addition, very little time is required. The maximum power level tested (10 kW generator power) also results in the thermal degradation of the material. Measurements were made up to about 1800 °C. However, the increase shows that temperatures in the range of over 2000 °C can be achieved if the determined heating rate is extrapolated according to the lower power levels.

**Figure 9:**
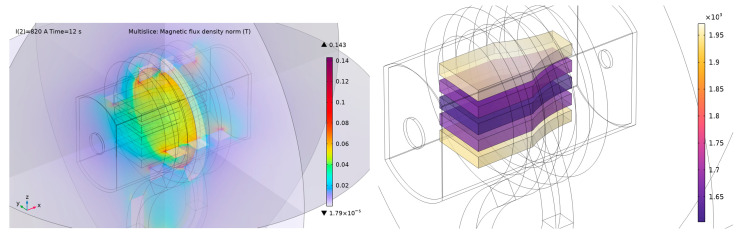
Magnetic flux density (**left**) and temperature distribution (**right**) on the combination of C/C-SiC specimens inside the ceramic shield.

These results can be used in further test series to work towards using the C/C-SiC as a material for a susceptor. Figure 9 shows results from a computer simulation made in COMSOL Multiphysics. We used the FEM method with the tetrahedral mesh type. In this case, we solved a coupled problem consisting of a harmonic electromagnetic field described by the Helmholtz equation for phasors

$$∆\underline{A}-j\omega \gamma \mu \underline{A}=-\mu {\underline{J}}_{ext}$$

and the temperature field described by the Fourier–Kirchhof equation

$$div\left(\lambda \;grad\;T\right)=\rho c_{p}\frac{\partial T}{\partial t}-w,$$

where

$$w=w_{J}+w_{H}$$

Hysteresis losses can be neglected, and Joule losses are considered to be

$$w_{J}=\frac{{\left|J_{ind}\right|}^{2}}{2\gamma },\;\;J_{ind}=-j\;\omega \gamma \mu \underline{A}.$$

Boundary conditions respecting convective and radiative heat flux are given by

$$-\frac{\partial T}{\partial n}=\alpha \left(T_{s}-T_{0}\right)+\sigma C\left(T_{s}^{4}-T_{r}^{4}\right).$$

These results show the possibility of using the C/C-SiC in such a kind of susceptor. The temperature distribution and magnetic flux density are depicted for a time of 12 s. For the frequency source, parameters from Table 2 were used: frequency f=260 kHz and power P=10 kW.

**Figure 10:**
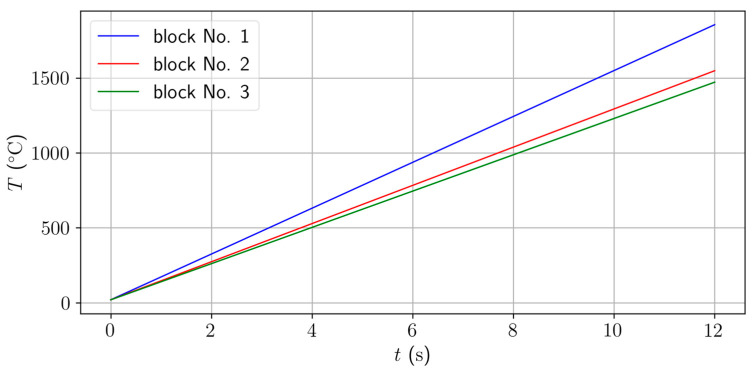
Time evolution of temperature in 3 upper blocks.

Figure 10 depicts the time evolution of temperature in three upper blocks. The presented results are comparable with the measurement—see Figure 4 and Figure 5.

This is capable of generating high temperatures locally and very quickly. For example, creating a porosity that heats a medium flowing through it would be necessary in industrial processes such as hot gas generation. A higher amount of porosity can lead to lower heating of the samples.

## 5. Conclusions

This research provides valuable insights into the thermal effects inducing electromagnetic waves. Through a combination of numerical FE simulations and experimental validations, the study comprehensively analyzes the distribution of the resulting temperature profiles within the carbon fibers. These findings offer crucial insights and lead to practical implications and limitations of the heating phenomena of several C/C SiC structures, facilitating advancements in high-temperature applications for industrial processes. The key parameters of the research approach lie in the design of the electromagnetic field in relation to the ceramic structure to be heated. If it is possible to place the comparatively long carbon fiber bundles in such a way that electrically conducting networks are formed by agglomeration, the investigated composite material will be particularly suitable for the high heating rates required. Linking the heating rate to the component design in a designed application allows the setting of different temperature fields and thus the control of process parameters in an industrial environment. This approach eliminates the need for complex temperature control of an entire system, which not only shortens the process chain, but is also more energy-efficient. The induction heating process is a new, innovative method of generating the process temperature by converting electrical energy into heat. Short-term, local overheating of the carbon fiber networks significantly increases the heating rate due to accelerated induction processes. The process for the inductive heating of C/C-SiC could be realized with the investigations carried out. Through selective heating, additional thermal stress on the system structure is minimized. The very short heating and cooling times validate the advantages of inductive heating. In the future, the use of efficient inductors in combination with porous cylindrical suspension pores should lead to significantly higher efficiencies; in addition, further research regarding the coil, generator, and process parameter optimization will be based on the presented results.

## Figures and Tables

**Table 1:** Frequencies corresponding to generator power.

Output Level	Generator Power [W]	Corresponding Frequency [kHz]	Depth of Penetration [mm]
1	500	175	12
2	2000	196	11
3	5000	220	10
4	7000	235	8
5		260	6

**Table 2:** Frequencies corresponding to generator power and different samples with the resulting hestin rate.

Generator Power [W]	Corresponding Frequency [kHz]	No.	CF Mass Share [%]	Maximum Temperature [°C]	Time [s]	Heating Rate [K/s]
500	175	1	CF40+10	911.1	48	9.36
		2	CF50	975.9	48	10.71
2000	196	3	CF40+10	1078.7	48	12.85
		4	CF50	1023.1	48	11.69
5000	220	5	CF40+10	1764.7	30.3	43.00
7000	235	6	CF50	1800.1	8.5	157.45
	260	7	CF40+10	1800.1	11.35	117.92
		8	CF50	1798.1	11.35	117.74

## Data Availability

The data presented in this study are available on request from the corresponding author. The data used to support the findings of this study are available from the paper.
